# Do Balloon Catheters have a Different Radial Force Along Their Longitudinal Axis?

**DOI:** 10.1007/s00270-024-03716-x

**Published:** 2024-04-19

**Authors:** Tae Won Choi, Jinoo Kim, Je Hwan Won

**Affiliations:** Department of Radiology, Ajou University School of Medicine, Ajou University Hospital, Suwon, Republic of Korea

**Keywords:** Angioplasty, Balloon catheter, Radial force

## Abstract

**Purpose:**

This experimental study was designed to compare radial forces between the central portion and both ends of balloon catheters when dilating stenosis.

**Materials and Methods:**

Three balloon catheters of 6 and 8 mm in diameter and of variable length were tested: Mustang, Conquest, and Genoss PTA. Cylindrical modules to position balloon catheters and install the measuring tip during radial force measurements were made using a 3D printer. The measuring tip created 20% stenosis at the inner lumen. Both ends and center of the balloon catheter were located at the measuring tip. The radial force was measured after inflating the balloon catheter to the rated burst pressure.

**Results:**

For the different diameters and lengths of balloon catheters and cylinder sizes, the median inccenter, the radial rease in radial force at the distal end compared to the center was 16.5% (range: 9.8–35.2%) for Mustang, 12.4% (range: 10.3–25.5%) for Genoss, and 7.4% (range: −0.3–13.1%) for Conquest balloon catheters. Similarly, compared to that at the force at the proximal end was 10.8% greater (range: −2.9–18.3%) for Mustang, 9.9% greater (range: 3.9–22.3%) for Genoss, and 7.3% greater (range: −1.3–12.4%) for Conquest catheters.

**Conclusion:**

The radial force is greater at both ends of the balloon than at the central portion, especially at the distal end. Dilation using the distal end of the balloon catheter is a practical method that can be applied in clinical practice without additional devices when encountering resistant stenosis, especially with semi-compliant balloons.

**Graphical Abstract:**

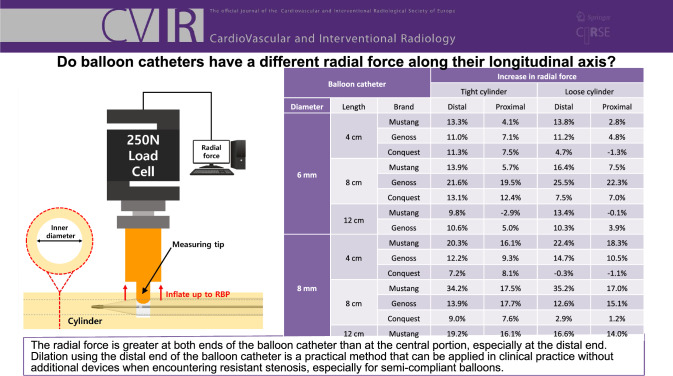

## Introduction

Even in the era of drug-eluting stents and drug-coated balloons, plain balloon catheters still play essential roles in angiography rooms, especially as a primary treatment modality for hemodialysis-related venous stenoses and a tool for vessel preparation in peripheral artery disease [1–4]. Vessel preparation is an essential step for creating a lumen with minimal injury to the vessel wall, usually followed by definitive therapy such as a drug-coated balloon or stent placement [5, 6]. Vessel preparation maximizes the luminal gain for stents and enhances drug uptake to the vessel wall, especially in heavily calcified lesions [5, 6].

However, some stenotic lesions are unresponsive to balloon dilatation, where persistent waist of the balloon is demonstrated despite being fully dilated up to its rated burst pressure (RBP) [7–9]. Dense calcification is the common cause of resistant stenoses unresponsive to balloon dilatation in peripheral artery disease [10, 11]. Resistant stenosis is known to be even more common in hemodialysis-related venous stenosis than in peripheral arterial disease [7, 12]. Trerotola et al. reported that 20% of native fistulas and 9% of grafts required balloon pressure higher than 20 atm to efface the balloon waist [13]. As a result of recurrent puncture trauma, dense fibrous strands may develop in the venous neointimal layer or scar tissue, causing resistant stenosis in hemodialysis-related venous stenoses [7, 9, 14]. In these cases, additional devices such as a non-compliant high-pressure balloon catheter or cutting balloon catheter may be required for successful dilatation of resistant stenoses [7, 9, 15]. However, the additional use of these specialized devices may pose cost-effectiveness issues and may not even be possible in some countries [9].

The radial force of the balloon catheter is known to depend on several factors, including the diameter and length of the balloon, inflation pressure, compliance of the balloon, and the degree and length of the stenotic lesion [16]. Although there are few previous studies on the mechanics of balloon catheter dilatation, several articles have suggested that the diameter of the balloon may be slightly different along its longitudinal axis [17, 18]. The literature also suggests that the thickness of the balloon catheter may not be uniform along its longitudinal axis because of the manufacturing process [19, 20]. We hypothesized that the radial force is different along the longitudinal axis when dilating resistant stenotic lesions. However, research on this phenomenon is lacking in the literature. Therefore, this experimental study was designed to compare radial forces between the central portion, and both ends of balloon catheters during balloon dilation.

## Materials and Methods

Three balloon catheters of 6 and 8 mm in diameter widely used in percutaneous transluminal angioplasty (PTA) procedures were tested: Mustang (Boston scientific, Natick, MA), Conquest (BD, Franklin lakes, NJ), and Genoss PTA (Genoss, Suwon, Korea). Mustang and Genoss are semi-compliant balloon catheters, and Conquest is a non-compliant balloon catheter [21–24]. The name of the manufacturer and size of balloon catheters and their nominal pressure and RBP experimented in this study are presented in Table 1.

**Table 1:** The brand and size of balloon catheters and their nominal pressures and rated burst pressure

Balloon catheter			Nominal pressure (atm)	Diameter (mm)	Rated burst pressure (atm)	Diameter (mm)
Diameter	Length	Brand				
6 mm	4 cm	Mustang	10	6.03	24	6.36
		Genoss	12	5.98	23	6.61
		Conquest*	8	5.78	40	5.97
	8 cm	Mustang	10	6.03	24	6.36
		Genoss	12	5.98	23	6.61
		Conquest*	8	5.71	40	5.97
	12 cm	Mustang	10	6.03	22	6.35
		Genoss	12	5.98	20	6.48
8 mm	4 cm	Mustang	10	8.05	20	8.46
		Genoss	12	7.99	18	8.28
		Conquest*	8	7.76	40	7.95
	8 cm	Mustang	10	8.05	20	8.46
		Genoss	12	7.99	18	8.28
		Conquest*	8	7.72	35	7.96
	12 cm	Mustang	10	8.05	18	8.38

*The authors measured the diameters of the Conquest balloon catheter at the nominal and rated burst pressure because of the lack of a detailed table provided by the manufacturer

This experimental study employed cylindrical modules made of transparent acrylic material to simulate the vessel wall. Because the transparent acrylic material has little elasticity and cannot simulate the elasticity of the vessel wall of the human, we experimented with two types of cylinders for each balloon diameter to simulate both extremes: tight and loose cylinders.

### Experiment 1: Tight Cylinders

**Fig. 1:**
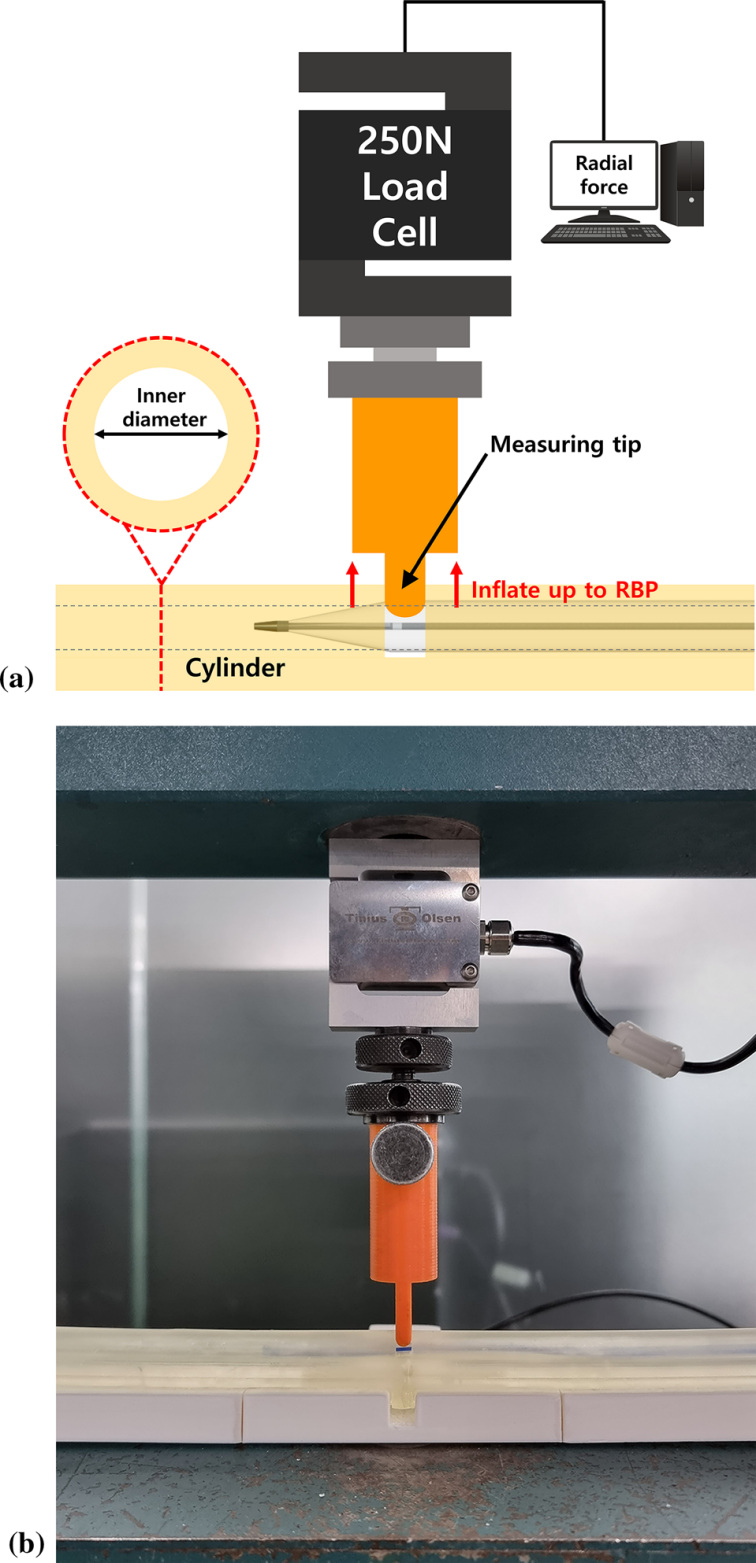
Schematic diagram (**a**) and pictures (**b**) of the experimental settings for measuring the radial force of the balloon catheter. The measuring tip was inserted through a hole made at the middle segment of the cylinder and was adjusted to create 20% stenosis at the inner lumen of the cylinder; the measuring tip was fixed to the load cell, where the magnitude of radial force was measured

Figure 1 demonstrates the design of cylindrical modules to position balloon catheters during radial force measurements and experimental setups. The cylindrical modules were designed using Solidworks 2015 software (Dassault Systemes, Vélizy-Villacoublay, France) and custom-made using a 3D printer (XFAB 2500SD, DWS system, Thiene, Italy) and transparent acrylic material (Vitra 413, DWS system).

The diameter of the tight cylinder was determined as 95% of the nominal diameter of the balloon catheter: 5.7 mm cylinder for 6 mm balloon catheters and 7.6 mm cylinder for 8 mm balloon catheters. When the balloon catheter was inflated to the RBP, the balloon surface came into full contact with the inner surface of the tight cylinder. The length of the cylindrical modules was 255 mm. A hole with a diameter of 5.2 mm was made at the middle segment of the cylinder to insert the measuring tip, which was made of polylactic acid and was 5 mm in diameter and rounded in shape.

We fixed the measuring tip to the DBBMTOL-250 N load cell (Tinius Olsen, Salfords, UK), where the magnitude of radial force was measured. The load cell and measuring tip were fixed to a testing machine (H50KT, Tinius Olsen), and the position of the measuring tip was adjusted to create 20% stenosis at the inner lumen of the cylinders.

The distal and proximal ends of the balloon’s tubular segment adjacent to the distal and proximal conical segments and the middle of the balloon’s tubular segment were located at the measuring tip, respectively. We fixed the balloon catheter in the cylinder to prevent axial movement during inflation. We used a balloon inflator device (B40, Genoss) with a rotating handle and pressure gauge and loaded it with normal saline to inflate the balloon catheters. The pressure was slowly increased by rotating the handle manually and under the monitoring of the pressure gauge to reach the RBP of the study devices. Thereafter, the load cell measured the radial force. All the measurements were repeated three times, and the median value was used for the results.

### Experiment 2: Loose Cylinders

The diameter of the loose cylinder was determined to be slightly larger than the diameter of the balloon catheters at the rated burst pressure: 6.7 mm cylinder for 6 mm balloon catheters and 8.5 mm cylinder for 8 mm balloon catheters (Table 1). When the balloon was inflated to RBP, the surface of the balloon did not contact the inner surface of the loose cylinder except for the measuring tip. The experimental setup, other than the diameter of the cylinder, was identical to that of Experiment 1.

## Results

Table 2 presents the radial force of the balloon catheter at the distal end, center, and proximal end when positioned inside the tight cylinder, and Table 3 presents the radial force inside the loose cylinders. For the different diameters and lengths of balloon catheters and cylinder sizes, the median increase in radial force at the distal end compared to the center was 16.5% (range: 9.8–35.2%) for Mustang balloon catheters, 12.4% (range: 10.3–25.5%) for Genoss balloon catheters, and 7.4% (range: −0.3–13.1%) for Conquest balloon catheters.

**Table 2:** The median radial force of the balloon catheter measured in the tight cylinder

Balloon catheter			Radial force (N)			Increase in radial force*	
Diameter	Length	Brand	Distal	Center	Proximal	Distal	Proximal
6 mm	4 cm	Mustang	22.1	19.5	20.3	13.3%	4.1%
		Genoss	23.3	21.0	22.5	11.0%	7.1%
		Conquest	29.5	26.5	28.5	11.3%	7.5%
	8 cm	Mustang	22.1	19.4	20.5	13.9%	5.7%
		Genoss	22.5	18.5	22.1	21.6%	19.5%
		Conquest	30.2	26.7	30.0	13.1%	12.4%
	12 cm	Mustang	22.5	20.5	19.9	9.8%	-2.9%
		Genoss	17.8	16.1	16.9	10.6%	5.0%
8 mm	4 cm	Mustang	31.4	26.1	30.3	20.3%	16.1%
		Genoss	27.6	24.6	26.9	12.2%	9.3%
		Conquest	47.7	44.5	48.1	7.2%	8.1%
	8 cm	Mustang	34.5	25.7	30.2	34.2%	17.5%
		Genoss	27.0	23.7	27.9	13.9%	17.7%
		Conquest	48.5	44.5	47.9	9.0%	7.6%
	12 cm	Mustang	31.1	26.1	30.3	19.2%	16.1%

*The force measured at the distal and proximal end of the balloon catheter was compared to the force measured at the center

**Table 3:** The median radial force of the balloon catheter measured in the loose cylinder

Balloon catheter			Radial force (N)			Increase in radial force*	
Diameter	Length	Brand	Distal	Center	Proximal	Distal	Proximal
6 mm	4 cm	Mustang	20.0	17.6	18.1	13.8%	2.8%
		Genoss	20.3	18.2	19.1	11.2%	4.8%
		Conquest	24.0	22.9	22.6	4.7%	-1.3%
	8 cm	Mustang	19.9	17.1	18.4	16.4%	7.5%
		Genoss	19.5	15.5	19.0	25.5%	22.3%
		Conquest	24.4	22.7	24.3	7.5%	7.0%
	12 cm	Mustang	20.7	18.3	18.2	13.4%	-0.1%
		Genoss	15.1	13.7	14.3	10.3%	3.9%
8 mm	4 cm	Mustang	28.7	23.5	27.7	22.4%	18.3%
		Genoss	23.4	20.4	22.6	14.7%	10.5%
		Conquest	37.9	38.1	37.6	-0.3%	-1.1%
	8 cm	Mustang	31.4	23.2	27.2	35.2%	17.0%
		Genoss	22.9	20.3	23.4	12.6%	15.1%
		Conquest	38.4	37.3	37.8	2.9%	1.2%
	12 cm	Mustang	27.8	23.8	27.1	16.6%	14.0%

*The force measured at the distal and proximal end of the balloon catheter was compared to the force measured at the center

For the different diameters and lengths of balloon catheters and cylinder sizes, the median increase in radial force at the proximal end compared to the center was 10.8% (range: -2.9–18.3%) for Mustang balloon catheters, 9.9% (range: 3.9–22.3%) for Genoss balloon catheters, and 7.3% (range: −1.3–12.4%) for Conquest balloon catheters.

Unlike the Mustang and Genoss balloon catheters, the Conquest balloon catheters had ratios of the radial force at both ends to the center that differed between the tight cylinder and the loose cylinder. For the distal end, the ratio decreased in the loose cylinder compared to the tight cylinder as follows: from 11.3 to 4.7% for the 6 mm–4 cm, from 13.1% to 7.5% for the 6 mm–8 cm, from 7.2 to −0.3% for the 8 mm–4 cm, and from 9.0 to 2.9% for the 8 mm–8 cm Conquest balloon catheter. For the proximal end, the ratio decreased in the loose cylinder compared to the tight cylinder as follows: from 7.5 to −1.3% for the 6 mm–4 cm, from 12.4 to 7.0% for the 6 mm–8 cm, from 8.1 to −1.1% for the 8 mm– 4 cm, and from 7.6 to 1.2% for the 8 mm–8 cm balloon catheter.

## Discussion

The results of the present study experimentally demonstrated that the radial force is greater at both ends of the balloon catheter than at the central portion, especially at the distal end. This phenomenon was more prominent for the semi-compliant balloon than for the non-compliant balloon. In clinical practice, we frequently encounter resistant stenosis unresponsive to balloon dilatation, especially when treating hemodialysis-related venous stenoses [7, 12, 13]. However, the use of a non-compliant high-pressure balloon as a first-line treatment in all cases is controversial because of its cost-effectiveness [9]. Based on the results of the present study, we suggest that when using semi-compliant balloons, dilation using the distal end of the balloon catheter is a practical method that can be applied in clinical practice without additional devices when encountering resistant stenosis.

**Fig. 2:**
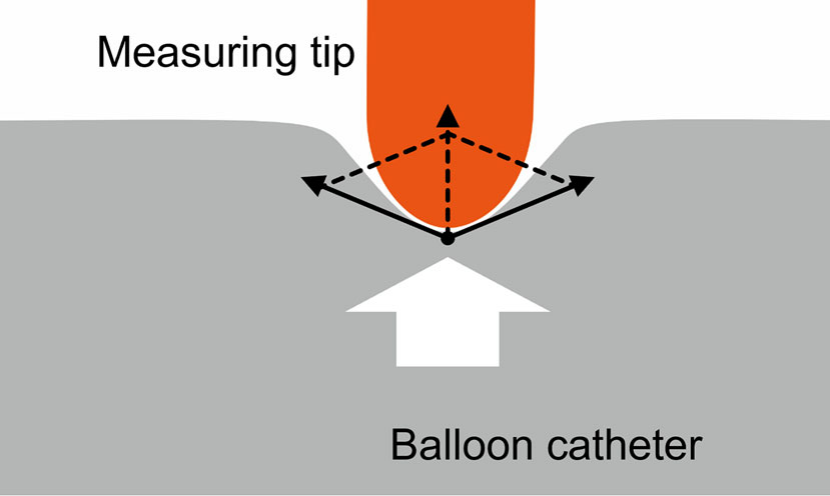
Schematic diagram showing the generation of radial force at the stenosis site during balloon dilatation. The radial force is determined by the sum of the pressure of saline inside the balloon (white arrow) and the tension of the balloon material (black arrows)

The following may explain why both ends of the balloon catheter produce greater radial force for stenosis. The radial force is determined by the sum of the pressure of saline inside the balloon and the tension of the balloon material, as illustrated in Fig. 2. Because the pressure of the saline in the end of the balloon is theoretically identical to that in the central portion, we speculate that the difference in the tension of the balloon material results in the difference in radial force between the two balloon locations. The tension of the distal or proximal portion may differ from that of the central portion because of the conical shape of the end and the different characteristics of the balloon material constituting the conically shaped end and cylindrically shaped central portion.

The ratio of the radial force at both ends to the center decreased in the loose cylinder compared to the tight cylinder for Conquest balloon catheters (Tables 2 and 3). This result presents a contrast with the Mustang and Genoss balloon catheters, where similar ratios were observed in tight and loose cylinders, and may be explained by the different characteristics of the Conquest balloon material as a high-pressure non-compliant balloon [7, 25]. Various materials are used to make balloon catheters and have different compliance-related properties. [20, 26, 27]. The balloon catheters experimented on in this study were constructed from a coextrusion of nylon and Pebax in Mustang catheters, nylon-12 in Genoss catheters, and ultrahigh molecular weight polyethylene in Conquest catheters [25, 28]. The difference in balloon material may have resulted in difference in tension and consequential radial forces in the present study (Fig. 2). The external compression caused by the elasticity and stiffness of the vascular wall is supposed to be in the middle ground between the conditions of our experiment using tight and loose cylinders. Therefore, the degree of increase in radial force at both ends of the Conquest balloon during the actual PTA procedure may also be in between the results of experiments using tight and loose cylinders. Overall, for non-compliant balloons, the technique of dilating resistant stenoses using the distal end of the balloon catheter may not be as effective as for semi-compliant balloons.

The phenomenon of greater radial force at both ends of the balloon catheter than at the central portion may seem similar to the so-called "dog-bone effect." The dog-bone effect refers to the phenomenon in which the balloon segment proximal and distal to the stenosis over-expands, especially when dilating resistant stenoses, and potentially poses a risk of vessel injury [25, 29]. Non-compliant balloons are known to show little dog-bone effect because they do not over-expand beyond their diameter due to the characteristics of the balloon material [25, 29]. However, the dog-bone effect described in the literature has often been associated with stenosis located at the middle segment of the balloon catheter during PTA. This differs from the experimental setting of the present study evaluating the radial force of both ends of the balloon catheter. In addition, the dog-bone effect in the balloon inflatable stent is not solely attributed to the characteristics of the balloon catheter. It can be understood as a phenomenon in which the mechanical properties of the balloon catheter and the mounted stent are combined [30, 31].

The following limitations of the present study should be noted. First, in this experiment, the balloon catheter was fixed in the cylinder to prevent axial movement during inflation. Second, as described above, because the cylinders simulating vascular walls are made of material with little elasticity, we experimented with two extreme settings using tight and loose cylinders. We also assumed that the stenosis was undilatable to all the balloons. The characteristics of the vascular wall and the presence of calcified lesions were not simulated. Therefore, the degree of increased radial force may differ in an actual vessel during PTA when compared to that in an experimental environment. Third, the design of the equipment used for this experiment simulated eccentric stenosis rather than concentric stenosis (Fig. 1). Further studies are needed to evaluate the radial force of the balloon when dilating concentric stenosis. The dedicated radial force testing machines used in the literature may be modified to simulate concentric stenosis and to measure the radial force [32–34]. Fourth, we tested only three balloon catheter brands frequently used in our institution. Therefore, one should be cautious when extrapolating the results of this study to other balloon brands.

## Conclusion

In conclusion, this experimental study demonstrated that the radial force is greater at both ends of the balloon catheter than at the central portion, especially at the distal end. Dilation using the distal end of the balloon catheter is a practical method that can be applied in clinical practice without additional devices when encountering resistant stenosis, especially with semi-compliant balloons.
